# Understanding the Contexts Where Clinical Supervision Is Implemented: An Interview Study

**DOI:** 10.1155/jonm/9963345

**Published:** 2025-08-20

**Authors:** Hosu Ryu, Bridget Hamilton, Lucio Naccarella, Niels Buus

**Affiliations:** ^1^Department of Nursing, Faculty of Medicine, Dentistry, and Health Science, Centre for Mental Health Nursing, University of Melbourne, Melbourne, Victoria, Australia; ^2^School of Nursing and Midwifery, La Trobe University, Melbourne, Victoria, Australia; ^3^Centre for Health Policy, Melbourne School of Population and Global Health, University of Melbourne, Melbourne, Victoria, Australia; ^4^Department of Public Health, Aarhus University, Aarhus, Denmark; ^5^School of Nursing and Midwifery, Monash University, Melbourne, Australia

**Keywords:** clinical supervision, mental health nursing, mentoring, organisations, working conditions

## Abstract

This qualitative study explores the contexts in which clinical supervision is implemented from the perspective of mental health nurses in Victoria, Australia. Clinical supervision has long been viewed as beneficial, offering the potential for increased job satisfaction, reduced stress and enhanced competencies. However, its implementation often faces challenges, with discrepancies between ideal outcomes and real-world supervision practices. Through semistructured interviews with 12 nurses, the study uncovers the complex dynamics influencing clinical supervision implementation, highlighting two key themes: (1) projecting hopes onto clinical supervision as a solution to systemic challenges, such as burnout and high staff turnover, and (2) noticing a disconnect between organisational emphasis on clinical supervision and the realities of the nursing work environment, especially facing a hierarchical culture that expects obedience and gratitude for minimal provision. For clinical supervision practice to be effective, it is essential to address the broader systemic and cultural challenges facing the nursing workforce through multiple solutions. Relying solely on clinical supervision, without addressing more fundamental underlying issues, will only perpetuate the challenges that clinical supervision aims to resolve. It also risks turning clinical supervision into a tool to shift the responsibility onto individual nurses to cope with systemic problems. Therefore, acknowledging the wider systemic challenges and demonstrating commitment to implementation through tangible support are essential to make clinical supervision truly meaningful for nurses.

## 1. Introduction

Clinical supervision is most often regarded as a beneficial practice for nurses, offering potential advantages, such as increased job satisfaction, staff retention, reduced stress, increased quality of care and more developed competencies [1, 2]. However, this practice continues to face scepticism, primarily due to the lack of consensus on its definition [3] and the varying strength of evidence supporting its benefits, as many studies that claim potential benefits rely mostly on self-reported qualitative methodologies based on a single site with modest sample size [3–5].

Efforts have been made to bridge a gap between promulgated ideals and the actual realities of these practices in the healthcare environment. Some studies suggest that the potential benefits asserted by the research community over the past 4 decades have not been fully realised due to the ineffective implementation of the practice [6]. Therefore, the focus has shifted from debating its benefits to exploring effective implementation of clinical supervision [2, 6, 7]. It is possible that the ideal-practice gap in clinical supervision does not stem from an inadequate understanding of the intervention itself but rather from insufficient attention to the implementation context, specifically, the environment to which this intervention is applied.

### 1.1. The Contexts Where Clinical Supervision Is Implemented

In implementation science, context is defined as “complex adaptive systems that form the dynamic environment in which implementation processes are situated” [8]. Without the understanding of the complex and dynamic environment in which clinical supervision implementation occurs, efforts may become ineffective, resources can be wasted and the supervision provided may not deliver intended benefits. When exploring the context of clinical supervision implementation, most of the literature identifies logistical difficulties such as shift work, available space and rostering as reasons for implementation challenges [9–14]. These studies also delve into the contexts of the nursing work, including workload, competing demands and difficulty finding time [9, 11, 15, 16], lack of human and material resources [17] and interruptions [10, 12]. Organisational culture, including levels of suspicion and resistance, is also explored [13, 14]. While these lists of barriers are helpful, we believe that richer discussions of the more ingrained assumptions of nursing work environments can enhance understanding of the factors driving behaviours that in turn impact clinical supervision practice and implementation.

In implementation studies, components that are considered normal conditions of the setting are rarely identified as confounding factors, despite their significant influence [8]. In other words, while tangible elements such as policies and management support are easily identified, research often does not delve into the basic underlying assumptions within the setting, including commonly held or shared belief perceptions and feelings, taken for granted within the organisation [18]. It is important to examine taken-for-granted assumptions, as these influence thinking and behaviour and thereby impact the process and outcome of implementation.

This study provides a nuanced description of dynamic environments where clinical supervision is implemented, from the perspective of mental health nurses. By addressing these deeper layers through the nurses' perspectives, this study seeks to go beyond identifying simple facilitators and barriers, providing a more insightful understanding of what influences the experience of clinical supervision for mental health nurses.

### 1.2. Aim

The aim of this study is to understand the context in which clinical supervision is implemented and practised, by exploring mental health nurses' perceptions of clinical supervision and its implementation in their work environment.

## 2. Methods

### 2.1. Design

This is a qualitative study using individual semistructured interviews.

### 2.2. Study Background

A scoping review of the clinical supervision implementation literature highlights a lack of policy direction in the systematic implementation of clinical supervision for nurses [7]. To address this issue, the Chief Mental Health Nurse from the Department of Health in Victoria, Australia, introduced a framework in 2018 to encourage a broader adoption of clinical supervision among Victoria's mental health nurses (Department of Health, 2018). While this framework was not prescriptive, it provided recommendations on how organisations could more widely implement this practice within their mental health nursing workforce. Detailing the specific implementation strategies applied is beyond the scope of this article; see Hamilton et al. [19].

This study reports the second, qualitative phase of a project exploring nurses' experiences with clinical supervision in environments where the framework was introduced, following a survey study of 422 mental health nurses [20], which detailed the characteristics of the supervision nurses are receiving. While this survey study was one of the largest investigations of the effectiveness of clinical supervision, it was subject to limitations inherent to survey research: response bias leading to challenges in generalisation [21] and a lack of depth and richness [22]. Therefore, this paper complements the quantitative analysis by exploring, in-depth, the nuanced experiences that the survey alone could not fully capture within their organisational context. This study does not evaluate the implementation outcomes; rather, it provides cross-sectional insights into nurses' experiences with clinical supervision and its implementation in their work environment 5 years after the clinical supervision policy framework introduction.

### 2.3. Participants and Recruitment

Participants were recruited from four services in Victoria, Australia, comprising three metropolitan and one rural service, from March to August 2023. These services are publicly funded and part of larger general healthcare system, offering a wide array of services including inpatient units, community services, and specialised mental health services. Nurses employed at these organisations were invited to participate in the aforementioned survey through posters and emails distributed by the nursing education team. At the conclusion of the survey, participating nurses were invited to leave email address if they would be willing to also participate in an interview. Data including participant information, recordings and transcripts were kept confidential, and anonymised at the transcription stage, ensuring that the interview participants were not identifiable. Eligible participants included any nurses currently receiving clinical supervision within these services.

Of those who expressed interest, 12 participants were invited for individual interviews, selected using demographic information (age, setting, experience, ethnicity, gender) to ensure a diverse sample. All 12 agreed to participate. The interviews concluded after these 12 participants, as data saturation was reached with no new insights emerging. Consequently, no further participants were invited for interviews.

The median age of the participants was 43, with a median of 15 years of experience as a nurse, and 6 years of experience in clinical supervision. Three participants were from culturally and linguistically diverse backgrounds. Ten participants currently received individual supervision, while two engaged in group supervision.

Further demographic data are presented in Table 1.

### 2.4. Interviews

For data collection, semistructured individual interviews were conducted via Zoom. The interview guide was developed based on the Consolidated Framework of Implementation Research (CFIR), which identifies determinants and variables influencing implementation effectiveness [23]. Participants were initially asked about their general experiences with clinical supervision, followed by questions related to each domain of the CFIR: intervention characteristics (in this case, supervision), outer setting, inner setting, characteristics of individuals and process. This approach aimed to explore participants' experiences within diverse implementation environments and contexts. The interview guide is attached as an appendix. The duration of the interviews ranged from 35 to 56 min, with an average of 50 min.

### 2.5. Data Analysis

The audio of the interviews was recorded using Zoom and saved on a password-protected university computer. The recordings were transcribed verbatim.

The data analysis followed the Braun and Clarke [24] approach to thematic analysis, which involves familiarisation with the data, generating initial codes, searching for and reviewing themes, and defining and naming themes [24]. One of the researchers (HR) coded the dataset using NVivo. This coding was guided by an open coding approach inspired by a grounded theory methodology, which codes for social–psychological processes, emphasising actions embedded within the codes, using gerunds, and making coding processes iterative [25]. To enhance the creativity and reliability of the analysis, another researcher (NB) independently coded two of the interviews and the two coders compared similarities and differences between their coding. Both researchers took notes during coding to strengthen reflexivity and gain a comprehensive understanding of each participant's perspectives. Once the data were fully coded, themes were generated by exploring recurring patterns and the relationships between codes [24]. All authors collaborated in reviewing the themes to refine them.

### 2.6. Ethical Considerations

The ethics application for this study was approved by the relevant health service ethics committee under the Australian National Mutual Acceptance (ID: 79097), and site-specific approvals were obtained at each participating site. The study was registered with the University of Melbourne Human Research Ethics Committee (ID: 2022-24598-29006-1).

Informed consent was obtained from each participant prior to the interview. Identifiable information was removed during the transcription stage and not reported in the manuscript. All participant names are pseudonyms. All files are stored on a password-protected computer for a minimum of 7 years, as per university policy, and will be securely disposed of after the retention period.

## 3. Results

We identified two overarching themes: (1) projecting hopes and (2) noticing a disconnect.  Table 2 provides a description of the theme and sub-themes and explains how they are related.

### 3.1. Theme 1—Projecting Hopes

The theme ‘projecting hopes' lays out what participants hoped to gain from clinical supervision practice and revealing what nurses perceived as lacking in their current work environment. In both these sub-themes, participants view clinical supervision as a tool for addressing a wide range of issues and concern. By engaging in clinical supervision, participants expressed hope to reconcile with the challenges in the current system but also to foster a sense of fulfilment in their work as mental health nurses.

#### 3.1.1. Reconciling With the Current Work Condition

Participants perceived clinical supervision as an activity that could reconcile them with the challenging conditions of their work. Nurses reported facing numerous difficulties, including high staff turnover, staff shortages, occupational violence and feelings of incompetency. Despite these challenges, participants expressed hope that clinical supervision could help alleviate their stress and mitigate the risk of burnout arising from these conditions.

Martin, a graduate nurse, had participated in a couple of group clinical supervision sessions so far in his practice and describes his supervision session:

We have our patient acuity increased. We had a rough… verbal and physical abuse and aggression. So quite a few of our staff are quite burnt out and emotionally quite taxing for us as well. So, then we had a bit of group supervision session on our shift that day. Then after the session, I specifically talk about the realities and stuff and some of the work issues. I felt quite motivated. Your work morale increased up a bit, because you had low mood and tired [before supervision] and after that you felt a bit energised.

While Martin saw clinical supervision as an activity that could help with staff morale, Nate, a nurse educator who both provided and received clinical supervision, also thought of this practice as a helpful way to manage stress that comes from nursing work. Nate stated:

I'm highlighting the burnout rate. Highlighting the value in doing supervision as well. How supervision can help empty that [stress] bucket and by emptying the bucket, giving you more skills, you don't feel so stressed, so overwhelmed.

While these participants recognised that work conditions such as short-staffing and other systemic issues were reasons for burnout, they expected that ‘emptying the bucket' through clinical supervision would prevent further burnout and sustain them, to work in the current conditions. Participants appeared inclined to focus inwardly on changing their reactions to workplace challenges, rather than to advocate for improvements in current work conditions from their organisations.

While recognising the current challenges of nursing workforce, Samantha, a senior manager, hoped that clinical supervision could be used as a retention tool for the organisation and the nursing workforce. Samantha said:

I think we use it [clinical supervision] as a retention tool because that will be one of the biggest things. So, I think if we can somehow show that this will help people stay in nursing.

Samantha's perspective as a senior manager introduces a strategic organisational viewpoint, hoping that clinical supervision could play a crucial role in retaining nursing staff, by supporting their well-being and demonstrating a commitment to their professional growth. This perspective also assumes that fostering shared reflection could help maintain nurses in a challenging work environment where retention is ‘one of the biggest things', evidently a difficult challenge. This highlights nurses' hope that clinical supervision could not only address individual stressors but also contribute to the broader organisational goals of workforce stability and sustainability.

#### 3.1.2. Protecting the Identity as a Mental Health Nurse

While some participants reported that stress and burnout from current work conditions were the main challenges in their workplace that clinical supervision could potentially solve, others viewed clinical supervision as a means to move beyond the task-oriented nature of nursing and ultimately to protect or expand their identity as mental health nurses. This sub-theme identified another hope projected on the clinical supervision practice, highlighting the participants' desire to integrate deeper meaning and personal fulfilment into their mental health nursing role and identity.

Jasmine, a clinical nurse who had been engaging in external clinical supervision for the last 10 years, saw clinical supervision as a meaning-making activity amidst the task-oriented nature of the work. Jasmine says:

Quite often in these community teams it can be really medication-based or physical health support based, and kind of come back to task orientated work. And I think it's really important to have that time where you're very much being yourself, being your nurse [sic], being in your discipline, and it's not about these kinds of really task based aspects, it's about that making meaning.

There appears to be discrepancies between how these participants expected the role of a mental health nurse to be and how they experienced it in practice. Charles, a clinical nurse who received external clinical supervision from a psychologist, also spoke to the tension between his ideal role of a mental health nurse and the often-limited scope of practice in reality. He stated that he did not ‘identify strongly with being a nurse' and that his role should be more related to psychological therapy, rather than traditional task-oriented nursing. Charles reflected on his desire for supervision as a place for skill development and stated:

Skill development I think is underplayed in nursing supervision. Or not even perhaps spoken about… That could be part of my views of what the role of a mental health nurse is and that they should be like able to provide high level, like advanced therapeutic skills in terms of psychological therapies and interventions. But then, nurses just provide medications and just need to be bodies on the ward type thing. Suppose I have goals that I want to use, achieve with clinical supervision, and a lot of that is around development of skills as a mental health nurse.

Charles believed that clinical supervision could enhance nurses' skills in providing what he perceived as more advanced therapies. In his account, there seemed to be a disconnect between what is expected of the nursing role and what he wanted to provide in the therapeutic environment. These participants saw the role of a mental health nurse as more than just ‘medication giving' and were trying to reconcile this discomfort through clinical supervision. This expectation reflected a desire for professional growth and validation in their profession as mental health nurses. They expressed a desire for clinical supervision to bridge the gap between their current experiences and their professional aspirations, seeking fulfilment in their identity, as mental health nurses. Additionally, other participants want this practice to help develop their clinical skills, foster resilience and improve their physical and mental health.

In this analysis, we see that participants had various expectations regarding what clinical supervision should provide for them and the nursing workforce, such as managing stress, improving clinical skills, helping with career progression and enhancing staff retention. However, these benefits did not necessarily reflect the actual outcomes of the current sessions. This theme highlighted how nurses project their hopes onto clinical supervision, which serves as a reflection of what they feel is lacking in their current work environment.3.2

### 3.2. Theme 2—Noticing a Disconnect

The second theme delves into participants' perceptions of their work environment in relation to their clinical supervision implementation. Rather than merely focussing on their immediate supervision experiences, they portray the nursing culture that was exposed through efforts to embed clinical supervision practices.

In the first sub-theme, participants explored how clinical supervision is practised within a professional culture perceived as hierarchical and controlling. The second sub-theme addresses participants' perceptions of a disconnect between the organisational intentions and the reality.

#### 3.2.1. Facing Hierarchical and Controlling Culture

This sub-theme explains how participants share their experiences of clinical supervision as it is organised in their work. They perceive that their profession within the organisation has a prevailing hierarchical and controlling culture and report how it interplays with the clinical supervision practices. Amelia, a clinical nurse working in a community team, was receiving clinical supervision from her line manager and reported her experience as ‘awful'. Amelia was reluctant to discuss the challenges she faces in her practice, fearing repercussions that could affect her job security and prospects for promotion, given that her supervisor had dual role, being a supervisor and a line manager. Amelia said:

There were times where the roles really got muddled. My nursing discipline senior would bring nursing-related tasks and ask me if I could do them during my supervision time, just because we were catching up.

She perceived a lack of clear boundaries in her sessions, being asked during the session by her manager or senior nurse to do certain things that were unrelated to the topic of supervision. She later added that she found it difficult to challenge these practices, as she believes that nursing culture is hierarchical.

This perception of nursing culture being hierarchical was shared by Jasmine. Jasmine reported receiving helpful supervision from an external supervisor, but in a new team, she was expected to receive clinical supervision from an internal supervisor as well. She reported attending the internal supervision to ‘appease' her workplace. Jasmine stated:

My service was like, ‘Oh no, we like clinical supervision, we think everyone should have supervision, but it has to be our way. And our way is we have, we know who's doing supervision with who, and we decide'. In terms of clinical supervision, ‘you'll be doing it with this senior nurse'. And I'm like, ‘I don't want do it with that senior nurse'. It became a little power struggle. It felt like it was insubordination.

Jasmine believed that the organisation's method of arranging supervision was driven by control and hierarchy. She voiced that the organisation's role in providing clinical supervision should not entail taking control of how and by whom the supervision is provided; however, she did not want to challenge this practice as she felt she was ‘being a bit disobedient'.

Both these participants shared that clinical supervision was treated as a line management space within their organisation, where they perceived it to be controlling and hierarchical. Amelia, however, added that this was not necessarily her organisational culture but rather a culture specific to nursing. While their organisation provided logistic support for clinical supervision, such as protected time and supervisors, they both reported that it did not necessarily translate into a meaningful and fulfilling experience for them. Participants described that the way clinical supervision was organised in their organisation contradicted the very essence of the practice.

#### 3.2.2. Not Expecting More

This sub-theme described how participants further shared the state of their current work environment, as they try to embed clinical supervision practices. They discussed the pressure to integrate clinical supervision without any additional resources, leaving nurses to manage within existing constraints. This sub-theme depicts the disconnect between the organisation's emphasis on clinical supervision and the practical resources allocated to realise it. Despite this evident disparity, participants were reluctant to advocate for further resources due to the prevailing nursing culture that expected gratitude for what was provided.

Teagan, a nurse educator in an inpatient unit, highlighted the severe staffing shortages that leave nurses unable to attend supervision sessions:

We're short-staffed, the health services are short-staffed everywhere. Nurses sometimes don't even take a break on the ward. There isn't any allocated time. We're with the patients the whole time. You're part of numbers. You cannot leave. I think nurses feel obligated to stay on the ward. They feel obligated to be there because they've always been part of the numbers.

Participants commonly report that the demanding workload, other training expectations and insufficient staffing left little room for nurses to participate in clinical supervision. The sense of obligation to remain on the ward as ‘part of numbers' and provide continuous care to patients overrides opportunities for clinical supervision. Another participant, Gemma, adds that attending clinical supervision is ‘a sacrifice' as they ‘go home late' if they come to supervision.

This demonstrates that nurses were not given additional staff or time to attend clinical supervision but were expected to fit it within existing resources, making it an additional burden. While participants recognise that their organisations wanted to implement clinical supervision, they wondered why there is such a discrepancy between the stated importance and the lack of follow-through and resources in practice. Sarah, an early career nurse, questioned this inconsistency. Sarah said:

It doesn't make sense to me that you would put it out there that this is important, this is what you need to do, or should do, it's beneficial, but then not actually take any steps to make sure it's happening. That doesn't, just doesn't add up. Like, why wouldn't you put it in the roster? I don't know. It would be something if it was actually important to them, surely?

While nurses were wondering about this disconnect, Jasmine shared that it was difficult to ask for more resources or support for supervision because she felt asking for more in nursing culture was not encouraged, and that nurses should be grateful for what they have. Jasmine said:

Sometimes it feels like your workplace sees it as they're being very progressive providing clinical supervision and that you're asking for more, and then that feels like that's the great crime in nursing ever is to not say thank you for what you're given and to ask for more is often seen as the greatest crime ever. We're just meant to take what we're given and say, ‘Thank you very much'.

Jasmine's words highlight the reluctance felt by nurses when advocating for better resources or support, indicating an entrenched expectation to be grateful for minimal provisions. This nuance can be interpreted as revealing the contradictions of a system that discourages those who seek to improve it, perpetuating a cycle of acceptance and minimal improvement.

In both sub-themes, participants described their working environments where clinical supervision is embedded and practised. They portrayed these environments as hierarchical and controlling, with scarce resources and an expectation of gratitude for minimal provision.

## 4. Discussion

This study explored the work environments where the clinical supervision is implemented. The first theme revealed insights into what nurses feel is lacking, leading them to project hope onto the practice of clinical supervision as a potential solution. The second theme highlighted the challenges within the work environment that they saw influencing the clinical supervision implementation.

The difficulty in implementing clinical supervision, due to the gap between its ideals and the cultural realities of nursing, has been previously explored [26]. While that early ethnographic study suggested that nurses perceive clinical supervision as having a limited experiential value, often seeking support from other formal and informal sources, our research adds a new dimension. We found that nurses highly value the idea of clinical supervision but see its effective implementation is hindered by the prevailing work environment condition.

First, participants' hopes for clinical supervision reflected the challenges they were currently facing in the workforce. These challenges align with the existing literature on nursing workforce, including understaffing [27, 28], high staff turnover [29, 30], stress and burnout [31, 32], workplace violence [33, 34], underutilisation of mental health nursing skills in practice [35] and perceived incompetence due to inadequate undergraduate preparation [36]. Participants implied an acceptance that these underlying issues might not be addressed, and that it was their responsibility to manage the systemic challenges, by adapting and changing their emotional reactions, by attending clinical supervision. This reliance on clinical supervision as a solution to various challenges reveals a gap in effective alternatives that can address core issues. Tony et al. [37] caution against over-reliance on clinical supervision and that the practice should not be seen as a ‘magic bullet' (p. 270) to numerous problems. We argue that clinical supervision should not be used as a tool to shift the burden of coping with inadequate work conditions onto individual nurses, nor should it give nurses false hope that this approach can resolve deeply rooted challenges on its own.

Second, our analysis highlighted that a hierarchical and controlling culture interferes with the implementation of meaningful clinical supervision. Nurses reported that requesting additional resources or improvements is often viewed as disobedience, as they are expected to be grateful for what is already provided. We propose that this issue may stem from the historical characteristics of the nursing workforce, which was predominantly made up of women. Calado Barbosa [38] suggested that societal expectations have historically created a dynamic in which women were expected to be obedient and grateful for the protection provided to them. Obedience was regarded not only as a duty but also as a morally virtuous trait. These historical cultural norms have shaped a nursing workforce culture [39] that emphasises acceptance and endurance over assertiveness and advocacy.

This challenge may be further exacerbated in modern nursing, particularly in countries like Australia, where the number of immigrant nurses is on the rise [40]. Immigrant nurses, some of whom participated in our study, may feel increased pressure to work under suboptimal conditions [41] and may encounter additional barriers when advocating for necessary resources [42]. We suggest that these societal expectations may have contributed to an environment where scarcity is accepted without question, perpetuating a cycle of inadequate support and ineffective implementation of clinical supervision.

Moreover, the interaction between the first theme, projecting hopes, and the second theme, noticing a disconnect, highlights a cyclical issue where the very challenges clinical supervision aims to address also hinder its effective implementation. For example, while clinical supervision is viewed as a solution to nursing shortages, these same shortages often prevent nurses from attending supervision sessions. Nurses are acutely aware of the entrenched challenges within their work environment, yet they continue to place hope in clinical supervision as a potential remedy. This creates a tension: while systemic issues create the need for clinical supervision, these same challenges also undermine its successful implementation. Nurses grapple with the paradox of placing hope in the intervention while accepting that the environment may not be capable of realising its full potential.

These disconnects between the intended benefits of supervision and its actual implementation underscore the need to understand and address systemic challenges within organisational environments. Failing to acknowledge the broader systemic challenges and treating clinical supervision as a separate component, independent of the complex environment in which it is implemented, risk using implementation resources ineffectively and undermining the intention behind the clinical supervision practice. Furthermore, demonstrating commitment through tangible support is essential, as allocating appropriate resources is integral to ensuring sustainability in implementation [43]. When organisational support for supervision is perceived as mere rhetoric rather than practical assistance, it can undermine the credibility of that assistance and risk eroding nurses' trust in both their profession and the organisations in which they work.

### 4.1. Strength and Limitation of the Study

The strength of this study lies in its in-depth exploration of the specific contexts in which clinical supervision is implemented. By exploring and challenging the underlying assumptions of these environmental contexts, the study offers insights on the deeper, systemic factors that influence the practice and perception of clinical supervision. This approach provides a more comprehensive understanding of the challenges faced in clinical supervision implementation and broader nursing workforce.

We do not claim this to be an evaluation of the implementation outcome, as we cannot establish a causal relationship between these experiences and the implementation. Instead, it offers cross-sectional insights into nurses' experiences of this practice and the work environment, 5 years after the clinical supervision policy framework was introduced to that environment.

Furthermore, while we aimed to interview nurses from diverse roles, including managers, educators and clinical nurses, we acknowledge that the sample is skewed towards education nurses. This trend was also identified in the first phase of the study [20], with two possible explanations. First, clinical supervision often falls under the education team's portfolio, making them more likely to engage as both supervisees and supervisors, which in turn increases their likelihood of volunteering to participate in the study. Second, our recruitment strategy may have contributed, as the education team disseminated the recruitment information, making its members more informed and more likely to participate. Despite this, we were able to obtain valuable perspectives from other roles, such as clinical nurses and managers, and meaningfully incorporate them into our analysis.

## 5. Conclusion

The novelty of the study lies in a nuanced exploration of the work environment where clinical supervision is implemented, embedded and practised. To ensure that clinical supervision is effective, it is essential to address broader systemic challenges the nursing workforce is facing through multiple solutions. Relying solely on clinical supervision, without addressing more fundamental underlying issues, will perpetuate the challenges that clinical supervision aims to resolve and will only add to the cyclical nature of the problem. While supporting the nursing workforce through clinical supervision may be beneficial, it must be approached with a clear understanding of its limitations and the broader context in which it operates. Without such consideration, clinical supervision may inadvertently become a tool to shift the responsibility onto individual nurses to cope with systemic problems, rather than a genuine and meaningful practice that meets the nurses' needs.

### 5.1. Relevance for Practice

Nurses' reliance on clinical supervision, typically a once-a-month reflective meeting, as a remedy for the multiple challenges they face suggests a lack of effective alternatives. While this study does not discount the benefits of clinical supervision, it highlights the need for critical reflection on the work environment in which it is implemented. Clinical supervision alone cannot resolve systemic challenges; only by addressing the underlying workforce issues through a multi-layered, concurrent approach can clinical supervision truly realise the hopes nurses have placed in it.

## Figures and Tables

**Table 1:** Participants' demographic information.

*Gender*	
Female/woman	7
Male/man	5

*Age*	
21–30	2
31–40	4
41–50	1
51–60	4
61–70	1

*Setting*	
Inpatient	6
Community	3
Neither or both	3

*Role*	
Clinical	6
Education	5
Managerial	1

**Table 2:** Themes and sub-themes.

Theme	Description	Sub-theme	Description
Projecting hopes	What participants perceive to be lacking in their current work environment was revealed through what they hope to achieve from the clinical supervision.	Reconciling with the current work condition.	Participants desiring clinical supervision to sustain and restore them, amidst the numerous difficulties they experience in the current work conditions.
		Protecting the identity of mental health nurse.	Participants desiring to integrate deeper meaning and personal fulfilment into their mental health nursing role and identity, through clinical supervision.

Noticing a disconnect	How participants perceive their work environment where the clinical supervision is being implemented.	Facing a hierarchical and controlling culture.	Participants perceiving the implementation of clinical supervision within a hierarchical and controlling culture as contradicting the very essence of the practice.
		Not expecting more.	Participants feeling reluctant to advocate for further resources, despite the perceived disconnect between the organisation's emphasis on clinical supervision and the limited practical resources allocated to realise its implementation.

## Data Availability

The data that support the findings of this study are available on request from the corresponding author. The data are not publicly available due to privacy or ethical restrictions.

## Appendix A. Semi-Structured Interview Guide

**CFIR Innovation characteristic**

• Tell me about your experience of receiving clinical supervision
• What did you expect from it? (potential questions)
• What do you do in your clinical supervision
• How do you spend your clinical supervision time?
• Were there any factors that influenced this experience, that you don't or didn't have any control over?

**CFIR Outer setting**

• Currently, there are a lot of conversation going on about clinical supervision in wider mental health nursing community, and also government support. Do you think these help the nurses to get clinical supervision? (Answer) Can you elaborate why you think that?
• What are the characteristics of your organisation that potentially help you and your colleagues from participating in clinical supervision?
• What are the characteristics of your organisation that potentially hinder you and your colleagues from participating in clinical supervision?

**CFIR Inner setting**

• How does your workplace culture influence your participation to clinical supervision?
• How do your workplace settings (inpatient, community etc.) influence your participation to clinical supervision?
• What are the characteristics of your unit/ immediate team that potentially help or hinder you or your colleagues from participating in clinical supervision?
• Do you think your workplace is ready for clinical supervision, why or why not?

**CFIR Characteristics of individual**

• How does your role (clinical, management, or educational) influence your participation to clinical supervision?
• Do you think nurses' personal attitude or knowledge affect their participation in clinical supervision, why or why not? Can you elaborate?

**CFIR Process**

• In your perspective, how do your nursing colleagues see the implementation of clinical supervision in your organisation? How do you see it?
• Was there a person who has been very helpful in implementing in clinical supervision in your organisation? Or someone who hasn't been very helpful? In what way?
• Do you think it's easy for your colleagues to find a supervisor, why or why not?

**Additional questions**

• Has anything got in the way of participating in supervision? Were there any other priorities?
• Is it easy for you to continue to participate in supervision, if yes, why, or it not, why not?
• Are you planning to continue to participate in supervision in the future, if yes, why, or if not, why not?
• Is there anything you would change in your clinical supervision? Can you tell me why?
• Imagine there are no external barriers to supervision, what would your ideal clinical supervision look like? (Following question: how is it different from right now?)
